# Incidence and clinical features of HHV-7 detection in lower respiratory tract in patients with severe pneumonia: a multicenter, retrospective study

**DOI:** 10.1186/s13054-023-04530-6

**Published:** 2023-06-23

**Authors:** Jun Xu, Lin Zhong, Huanzhang Shao, Qianqian Wang, Muhua Dai, Peng Shen, Yonghui Xiong, Weijun Zhang, Xutao Deng, Mingqiang Wang, Yue Zhu, Xindie Reng, Yongpo Jiang, Mengyuan Chen, Chengcong Zhu, Xueling Fang, Guojun He, Yijiao Han, Xiaohan Huang, Xuwei He, Yinghe Xu, Hongliu Cai, Lingtong Huang

**Affiliations:** 1 Department of Critical Care Medicine, The First Affiliated Hospital of Zhejiang University, Hangzhou, China; 2 Department of Critical Care Medicine, The First People’s Hospital of Pinghu, Pinghu, China; 3 Department of Critical Care Medicine, Henan Provincial People’s Hospital, Zhengzhou University People’s Hospital, Henan University People’s Hospital, Zhengzhou, China; 4 Department of Critical Care Medicine, The First Hospital of Jiaxing, Jiaxing, China; 5 Department of Critical Care Medicine, Tongde Hospital of Zhejiang Province, Hangzhou, China; 6 Department of Critical Care Medicine, Lanxi Hospital of Traditional Chinese Medicine, Lanxi, China; 7 Department of Critical Care Medicine, Lishui People’s Hospital, Lishui, China; 8 Department of Critical Care Medicine, Taizhou Hospital of Zhejiang Province Affiliated to Wenzhou Medical University, Taizhou, China; 9 Department of Respiratory Care, The First Affiliated Hospital, Zhejiang University School of Medicine, Hangzhou, China; 10 Kidney Disease Center, The First Affiliated Hospital of Zhejiang University, Hangzhou, China

## Abstract

**Purpose:**

The significance of detecting human herpesvirus 7 (HHV-7) in the lower respiratory tract of patients with severe pneumonia is unclear. This study aims to evaluate the clinical characteristics and prognosis of detecting HHV-7 in the lower respiratory tract of patients with severe pneumonia.

**Methods:**

Patients with severe pneumonia requiring invasive mechanical ventilation and underwent commercial metagenomic next-generation sequencing (mNGS) testing of bronchoalveolar lavage fluid from January 2019 to March 2023 were enrolled in 12 medical centers. Clinical data of patients were collected retrospectively, and propensity score matching was used for subgroup analysis and mortality assessment.

**Results:**

In a total number of 721 patients, 45 cases (6.24%) were identified with HHV-7 positive in lower respiratory tract. HHV-7 positive patients were younger (59.2 vs 64.4, *p* = 0.032) and had a higher rate of co-detection with Cytomegalovirus (42.2% vs 20.7%, *p* = 0.001) and Epstein–Barr virus (35.6% vs 18.2%, *p* = 0.008). After propensity score matching for gender, age, SOFA score at ICU admission, and days from ICU admission to mNGS assay, there was no statistically significant difference in the 28-day mortality rate between HHV-7 positive and negative patients (46.2% vs 36.0%, *p* = 0.395). Multivariate Cox regression analysis adjusting for gender, age, and SOFA score showed that HHV-7 positive was not an independent risk factor for 28-day mortality (HR 1.783, 95%CI 0.936–3.400, *p* = 0.079).

**Conclusion:**

HHV-7 was detected in the lungs of 6.24% of patients with severe pneumonia. The presence of HHV-7 in patients with severe pneumonia requiring invasive mechanical ventilation is associated with a younger age and co-detected of Cytomegalovirus and Epstein–Barr virus. While HHV-7 positivity was not found to be an independent risk factor for mortality in this cohort, this result may have been influenced by the relatively small sample size of the study.

**Supplementary Information:**

The online version contains supplementary material available at 10.1186/s13054-023-04530-6.

## Introduction

Herpesvirus in the intensive care unit (ICU) has been widely reported, including cytomegalovirus, herpes simplex virus, and Epstein–Barr virus, among others [1–5]. However, few studies have reported on the clinical features of human herpesvirus 7 (HHV-7) positive in the lower respiratory tract of patients in the ICU. HHV-7 is a member of beta-herpesvirus subfamily and was discovered in the late twentieth century [6, 7]. HHV-7 infection is common and more than 95% of the adults are serologically positive for HHV-7 [8]. While the virus is ubiquitous, its significance in human diseases is unclear. Detection of HHV-7 in the lungs has been identified as a risk factor for interstitial pneumonia or idiopathic pulmonary fibrosis [9, 10]. Some studies have described the detection of HHV-7 in the bronchoalveolar lavage fluid (BALF) of patients with ARDS or chronic bronchopulmonary diseases, but these studies have not adequately described the clinical characteristics due to the small sample size [11, 12].

The incidence, clinical characteristics, and significance of HHV-7 detected in the lower respiratory tract of patients with severe pneumonia have not been well described. Here, we evaluated the clinical characteristics and prognosis of HHV-7 positive in patients with severe pneumonia through a multicenter retrospective study.

## Methods

### Patients and data collection

This multicenter retrospective cohort study was conducted in adult ICUs with approximately 800 ICU beds in 12 medical centers in China. The study has been approved by the ethics committees of all participating hospitals.

Patients with severe pneumonia requiring mechanical ventilation and underwent commercial metagenomic next-generation sequencing (mNGS) testing of BALF from January 2019 to March 2023 were enrolled. The mNGS testing laboratory was certified by either the College of American Pathologists or the External Quality Assessment program of the Chinese National Health Commission. Exclusion criteria were: 1. age less than 18 years; 2. mNGS testing performed more than 28 days after ICU admission; and 3. loss to follow-up within 28 days after ICU admission. Data were collected that included age, gender, comorbidities, and sequential organ failure assessment (SOFA) score at ICU admission or mNGS detected day. The definition of immunosuppression is the same as previously described [1]. The missing data were imputed using multiple imputation.

### Data analysis and propensity score matching

Student’s *t* test for the continuous variables and Chi-square test or Fisher’s exact test for the categorical variables were used. Propensity score matching analysis was conducted with the 1:2 optimal matching method and a caliper width of 0.02 by the “MatchIt” package in R software to establish a balance in baseline characteristics between HHV-7 positive group and HHV-7 negative groups. Kaplan–Meier survival curves and subgroup analysis were used to compare the differences in mortality between the two groups. In the matched cohort, a multivariate Cox regression model was used to identify independent risk factors for 28-day mortality. The included factors were gender, age, positive HHV-7 status, SOFA score, and parameters with *p* < 0.1 in univariate analysis. All statistical analyses were performed using R software (v4.2.3) and *p* < 0.05 (two-tailed) were considered significant. Drawing Sankey diagram of species detected in BALF was performed by using Pyecharts 2.0.3 library through Python 3.11.3.

### Sensitive analysis

Sensitive analysis was performed. 28-day mortality after mNGS testing was reported in this study.

## Results

A total of 872 patients were enrolled in this study. After excluding patients who met the exclusion criteria, 721 patients were included in the analysis. Among them, HHV-7 was detected in the lungs of 45 patients (6.24%), while HHV-7 was not detected in the lungs of 676 patients (93.76%) (Additional file 1: Fig. S1). HHV-7 positive patients were younger than negative patients (59.2 vs 64.4, *p* = 0.032), but there were no significant differences in gender and baseline comorbidities between the two groups (Table 1). In addition, patients with HHV-7 detected in the lungs had a higher incidence of other herpesviruses than those without (71.1% vs 50.3%, *p* = 0.011), such as Epstein–Barr virus (35.6% vs 18.2%, *p* = 0.008) and Cytomegalovirus (42.2% vs 20.7%, *p* = 0.001). Regarding prognosis, there was no significantly difference in the total length of hospital stay (30.4 vs 30.7, *p* = 0.942) and 28-day mortality after admission to ICU (40.2% vs 40%, *p* = 1).

**Table 1:** Characteristics of invasive mechanically ventilated patients with or without HHV-7 lung detection in the original cohort and the propensity score matching cohort

Variables	Original cohort			Matched cohort		
	HHV-7 negative (*n* = 676)	HHV-7 positive (*n* = 45)	*P*-value	HHV-7 negative (*n* = 75)	HHV-7 positive (*n* = 39)	*P*-value
Age, mean (SD)	64.4 (15.5)	59.2 (19.0)	0.032	65.7 (14.7)	64.3 (14.7)	0.630
Male, *n* (%)	470 (69.5)	37 (82.2)	0.102	62 (82.7)	31 (79.5)	0.872
Comorbidities, *n* (%)						
Diabetes mellitus	151 (22.3)	7 (15.6)	0.380	11 (14.7)	7 (17.9)	0.853
Congestive heart failure	80 (11.8)	7 (15.6)	0.613	9 (12.0)	6 (15.4)	0.830
Myocardial infarction	30 (4.4)	2 (4.4)	1.000	2 (2.7)	2 (5.1)	0.888
Chronic pulmonary disease	118 (17.5)	8 (17.8)	1.000	13 (17.3)	8 (20.5)	0.872
Liver disease	50 (7.4)	1 (2.2)	0.312	5 (6.7)	1 (2.6)	0.625
Renal disease	68 (10.1)	5 (11.1)	1.000	6 (8.0)	3 (7.7)	1.000
Solid tumor	106 (15.7)	5 (11.1)	0.542	10 (13.3)	5 (12.8)	1.000
Hematological malignancy	36 (5.3)	2 (4.4)	1.000	4 (5.3)	2 (5.1)	1.000
CTD	28 (4.1)	2 (4.4)	1.000	3 (4.0)	2 (5.1)	1.000
Transplantation	29 (4.3)	2 (4.4)	1.000	2 (2.7)	2 (5.1)	0.888
CAP, *n* (%)	310 (45.9)	16 (35.6)	0.234	39 (52.0)	15 (38.5)	0.240
Immunosuppressive status, *n* (%)	175 (25.9)	12 (26.7)	1.000	20 (26.7)	11 (28.2)	1.000
SOFA score at ICU admission, mean (SD)	8.0 (3.5)	7.8 (3.1)	0.637	8.6 (3.4)	7.7 (3.1)	0.193
Respiratory score	2.5 (1.1)	2.6 (1.0)	0.551	2.7 (1.0)	2.7 (0.9)	0.892
Coagulation score	1.0 (1.2)	0.8 (1.2)	0.507	1.1 (1.3)	1.0 (1.2)	0.713
Liver score	0.5 (0.9)	0.4 (0.9)	0.425	0.7 (1.1)	0.4 (0.7)	0.128
Cardiovascular score	1.7 (0.9)	1.3 (0.8)	0.003	1.7 (0.9)	1.3 (0.9)	0.027
Neurological score	1.7 (1.6)	2.0 (1.7)	0.234	1.8 (1.7)	2.0 (1.7)	0.663
Kidney score	0.7 (1.2)	0.7 (1.2)	0.979	0.7 (1.1)	0.5 (1.0)	0.386
SOFA score at NGS time, mean (SD)	9.2 (3.2)	9.4 (3.1)	0.649	9.3 (3.3)	9.5 (3.3)	0.784
Respiratory score	3.1 (0.3)	3.1 (0.3)	0.622	3.1 (0.3)	3.2 (0.4)	0.767
Coagulation score	1.2 (1.2)	1.2 (1.3)	0.804	1.2 (1.2)	1.3 (1.3)	0.591
Liver score	0.6 (1.0)	0.5 (0.9)	0.838	0.7 (1.1)	0.5 (0.8)	0.297
Cardiovascular score	1.8 (0.9)	1.7 (0.9)	0.333	1.7 (1.0)	1.7 (0.9)	0.793
Neurological score	1.8 (1.6)	2.0 (1.7)	0.360	2.0 (1.7)	2.2 (1.7)	0.508
Kidney score	0.7 (1.2)	0.8 (1.3)	0.602	0.7 (1.2)	0.7 (1.1)	0.859
Days from transfer to ICU to mNGS assay, mean (SD)	5.7 (5.6)	5.7 (5.7)	0.974	5.0 (4.8)	5.2 (5.2)	0.791
Herpesviridae, *n* (%)	340 (50.3)	32 (71.1)	0.011	35 (46.7)	27 (69.2)	0.036
Herpes simplex virus-1	206 (30.5)	15 (33.3)	0.813	15 (20.0)	13 (33.3)	0.180
Varicella zoster virus	5 (0.7)	0 (0.0)	1.000	0 (0)	0 (0)	NA
Epstein–Barr virus	123 (18.2)	16 (35.6)	0.008	15 (20.0)	13 (33.3)	0.180
Cytomegalovirus	140 (20.7)	19 (42.2)	0.001	23 (30.7)	15 (38.5)	0.530
Human herpesvirus-6B	12 (1.8)	2 (4.4)	0.485	1 (1.3)	1 (2.6)	1.000
Hospital LOS, mean (SD)	30.4 (29.3)	30.7 (24.0)	0.942	26.8 (21.3)	28.3 (23.0)	0.728
28-day mortality, *n* (%)	272 (40.2)	18 (40.0)	1.000	27 (36.0)	18 (46.2)	0.395

*ICU*, intensive care unit; *SD*, standard deviation; *MI*, myocardial infarction; *CTD*, connective tissue disease; *CAP*, community-acquired pneumonia; *SOFA*, sequential organ failure assessment; *NGS*, next-generation sequencing; *LOS*, length of stay

**Fig. 1:**
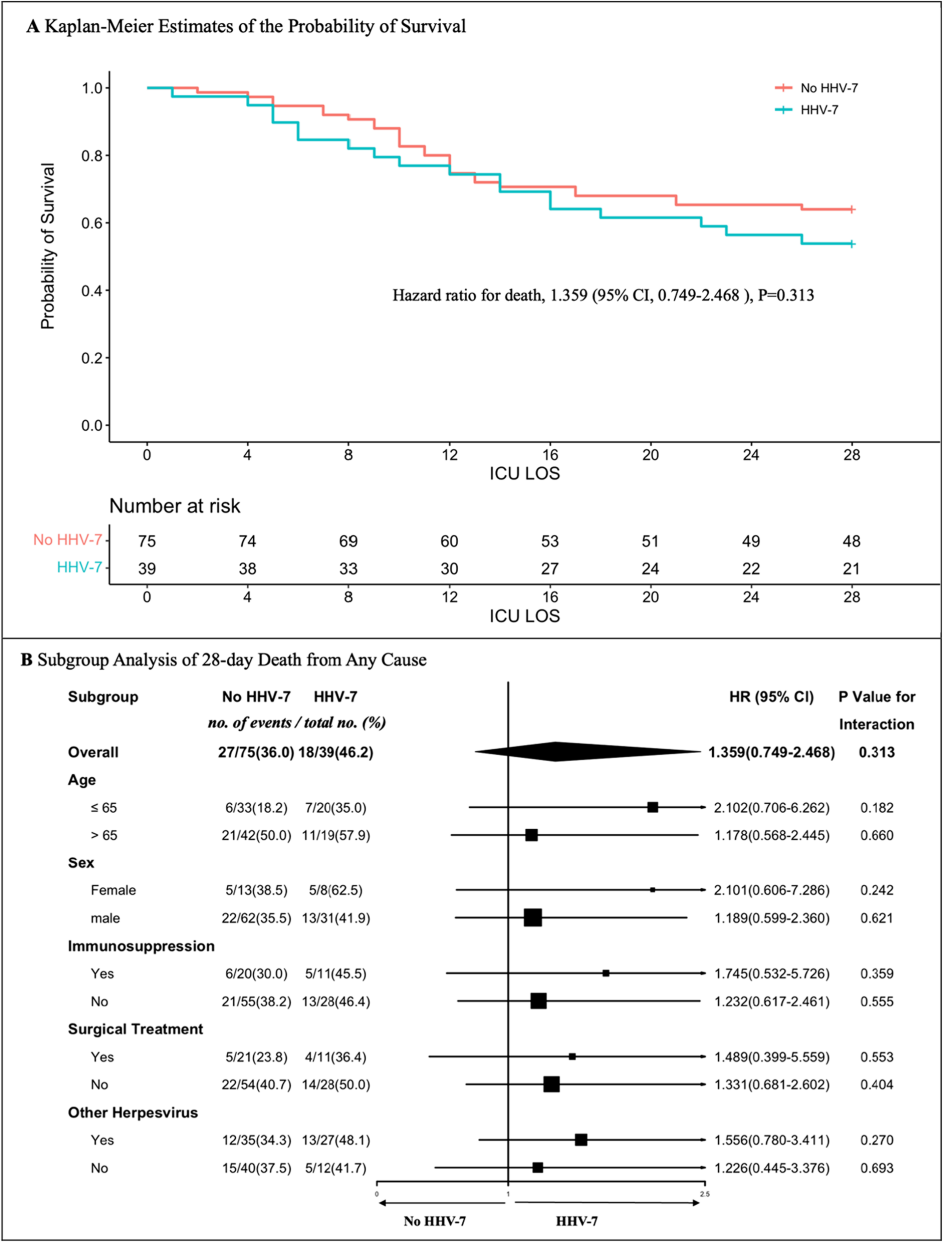
**A** Kaplan–Meier curve of Propensity score matching cohort. **B** Subgroup analysis of 28-day death from any cause

Propensity score matching was performed with gender, age, SOFA score at ICU admission, and time from ICU admission to mNGS testing. The 28-day mortality in HHV-7 positive group was higher than HHV-7 negative group (46.2% vs 36.0%) without statistically significant difference (*p* = 0.395). No statistical difference was also observed between the two groups based on the Kaplan–Meier survival curve analysis (*p* = 0.313) (Fig. 1A). In subgroup analysis, no statistical difference in mortality rate was observed between the two groups of patients (Fig. 1B). After adjusted for gender, age, SOFA score, and the presence of other herpesviruses, the results of cox multivariate regression analysis showed that HHV-7 was not an independent risk factor for 28-day mortality (HR 1.783, *p* = 0.079) (Additional file 2: Fig. S2). Finally, 28-day all-cause mortality after mNGS testing in sensitivity analyses showed no statistically significant difference between the two groups (51.3% versus 40% *p* = 0.263) (Additional file 3: Fig. S3A). Besides, no statistically significant difference was observed in subgroup analysis, but HR was greater than 1 in all subgroups (Additional file 3: Fig. S3B). Finally, we present the main microbial pathogens within BALF in HHV-7-positive or HHV-7-negative patients detected by mNGS (Additional file 4: Fig. S4).

## Discussion

In this study, we assessed the incidence and clinical characteristics of HHV-7 in the lower respiratory tract of patients with severe pneumonia. As an unbiased detection method, mNGS has been widely used for pathogen identification [13]. In this study, mNGS of bronchoalveolar lavage fluid (BALF) was used to determine the incidence of HHV-7 in the lungs and whether it was co-detected with other herpes viruses. In our study, 6.24% of BALF was positive for HHV-7. The previous prospective observational studies have shown that HHV-7 was associated with younger age which is consistent with the results we have observed [14]. About 71.1% of HHV-7 positive patients were also detected with other herpes viruses, which is significantly higher than the HHV-7 negative group. The reactivation of different viruses in the lungs may have different patterns. The detection of HSV-1 in the lungs is believed to be associated with viral reactivation in the mouth or throat [15]. HHV-7 could also be detected in the mouth, but many studies have shown that reactivation of HHV-7 is also observed in lung detection which means that HHV-7 in the lungs may not be caused by aspiration of saliva [9, 11, 16]. In HHV-7 positive group, two patients had been on long-term anti-rejection drugs following previous kidney transplantation, one patient received rituximab treatment for diffuse large B cell lymphoma three weeks prior to the onset of the disease, and the remaining patients with immunosuppression in the HHV-7 positive group were undergoing long-term maintenance therapy with low-dose methylprednisolone for connective tissue disease or other reasons. Only 26.7% of HHV-7 positive patients had clear evidence of immunosuppression, which means that HHV-7 may be as widely present in non-immunosuppressed patients as other herpes viruses [1].

The treatment of HHV-7 remains controversial. The prophylactic dose of acyclovir seems unable to prevent the reactivation of HHV-7 [17], while the antiviral drug cidofovir used for the treatment or prevention of HHV-7 has serious side effects [18]. If it is clear that the reactivation of HHV-7 is pathogenic in critically ill patients, then developing new broad-spectrum anti-herpes virus drugs is the direction of future research.

This study has several limitations. Firstly, it is a retrospective study, which introduces a certain degree of selection bias. Secondly, our study only reported a 6.24% prevalence of HHV-7 in patients, which may limit the statistical power of the study. Thirdly, the accuracy of mNGS may not be as precise as polymerase chain reaction testing. It has been reported that high viral load is associated with adverse clinical outcomes [5, 19]. However, due to the limitations of mNGS in quantitatively detecting HHV-7 viral load, we were unable to analyze the clinical characteristics of patients with high HHV-7 viral load positivity. Lastly, this is a cross-sectional study, so we cannot confirm whether the detection rate of HHV-7 increases with prolonged hospital stay, which is observed in other herpesviruses [20]. However, our study also has its strengths. It is a multicenter study with an adequate number of included patients, and the unbiased mNGS technique was used to detect herpesviruses throughout the entire sample, allowing us to observe the association between HHV-7 and other herpesviruses being detected simultaneously. At last, the detection rates of HSV-1 and CMV reported in our study are similar to those described previously [2, 5], which indicated that our detection rate of HHV-7 should be accurate.

## Conclusion

HHV-7 was detected in the lungs of 6.24% of patients with severe pneumonia. The presence of HHV-7 in patients with severe pneumonia requiring invasive mechanical ventilation is associated with a younger age and co-detected of Cytomegalovirus and Epstein–Barr virus. While HHV-7 positivity was not found to be an independent risk factor for mortality in this cohort, this result may have been influenced by the relatively small sample size of the study.

## Supplementary Information

**Additional file 1**: **Fig S1**. Study profile.

**Additional file 2**: **Fig. S2**. The results of multivariable analyses for 28-day all-cause mortality with the Cox regression model, *p* < 0.05 were considered statistically significant and shown in bold.

**Additional file 3**: **Fig. S3**. Sensitive analysis.

**Additional file 4**: **Fig. S4**. Main microbial pathogens including bacteria, virus, fungi, and others detected by mNGS within HHV-7-positive and HHV-7-negative BALF. All data represent the number of times pathogens were detected within different groups, not the number of patients.

## Data Availability

The data can be obtained from the corresponding author LTH upon reasonable request.
