# Supplementary material for: Incidence and clinical features of HHV-7 detection in lower respiratory tract in patients with severe pneumonia: a multicenter, retrospective study
Source: Crit Care. 2023 Jun 23;27:248. doi: 10.1186/s13054-023-04530-6 (PMC10290302; doi:10.1186/s13054-023-04530-6)
Supplement: Supplementary file 1 — Additional file 1: Fig S1. Study profile. [file 13054_2023_4530_MOESM1_ESM.pdf]

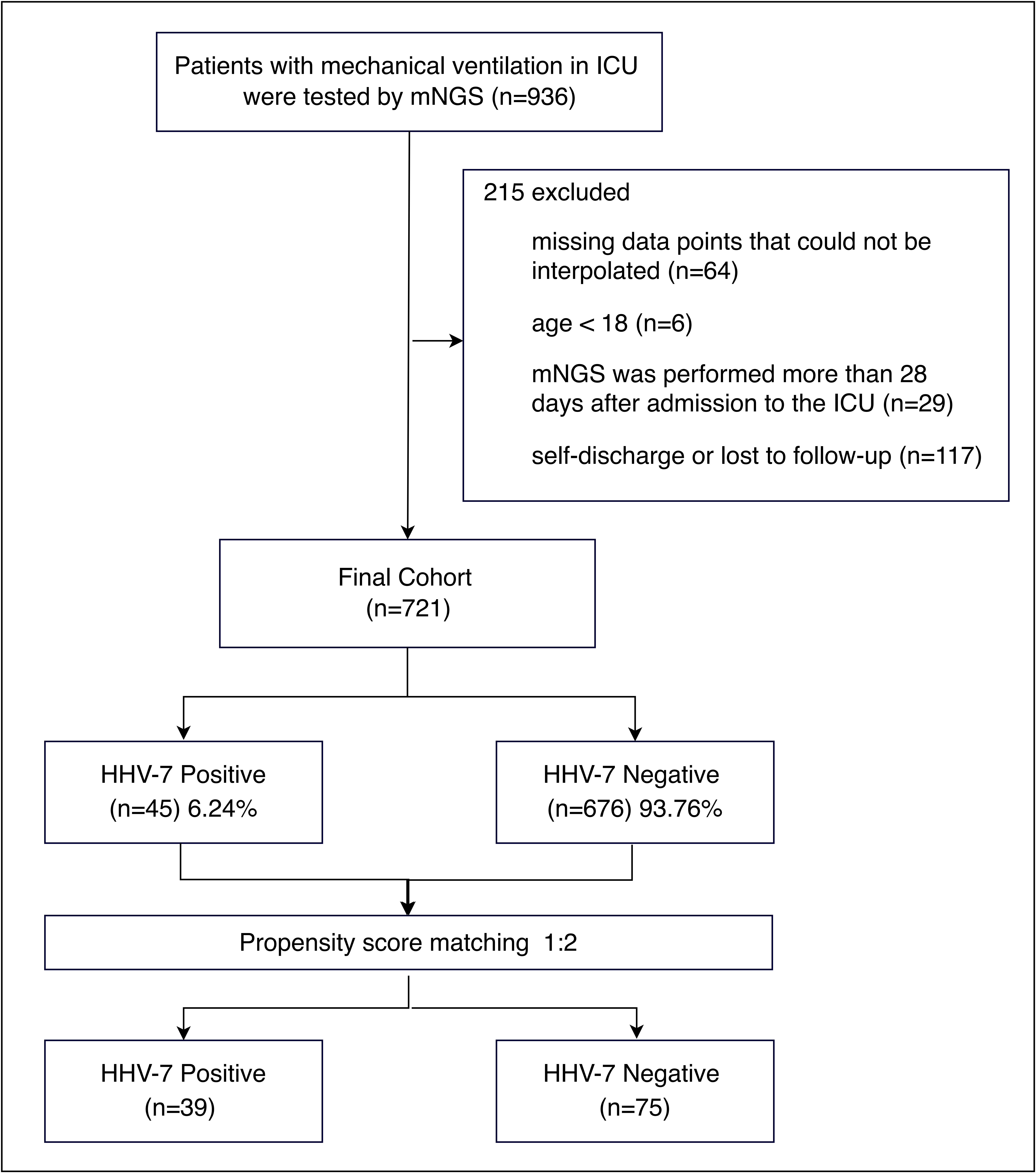
Patients with mechanical ventilation in ICU were tested by mNGS (n=936)
215 excluded
missing data points that could not be interpolated (n=64)
age < 18 (n=6)
mNGS was performed more than 28 days after admission to the ICU (n=29)
self-discharge or lost to follow-up (n=117)
Final Cohort (n=721)
HHV-7 Positive (n=45) 6.24%
HHV-7 Negative (n=676) 93.76%
Propensity score matching 1:2
HHV-7 Positive (n=39)
HHV-7 Negative (n=75)
