# Supplementary figures and images for: Incidence and clinical features of HHV-7 detection in lower respiratory tract in patients with severe pneumonia: a multicenter, retrospective study
Source: Crit Care. 2023 Jun 23;27:248. doi: 10.1186/s13054-023-04530-6 (PMC10290302; doi:10.1186/s13054-023-04530-6)

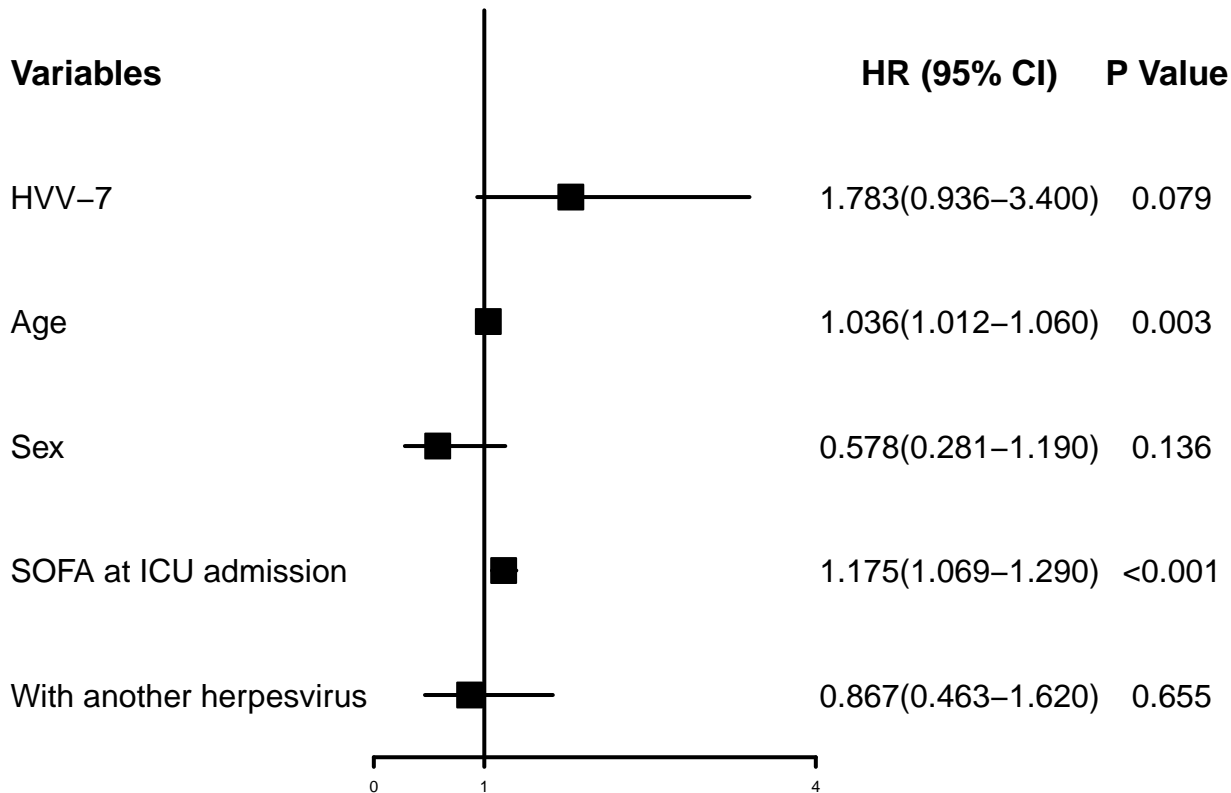

Supplement: Supplementary file 2 — Additional file 2: Fig. S2. The results of multivariable analyses for 28-day all-cause mortality with the Cox regression model, p < 0.05 were considered statistically significant and shown in bold. [file 13054_2023_4530_MOESM2_ESM.pdf]

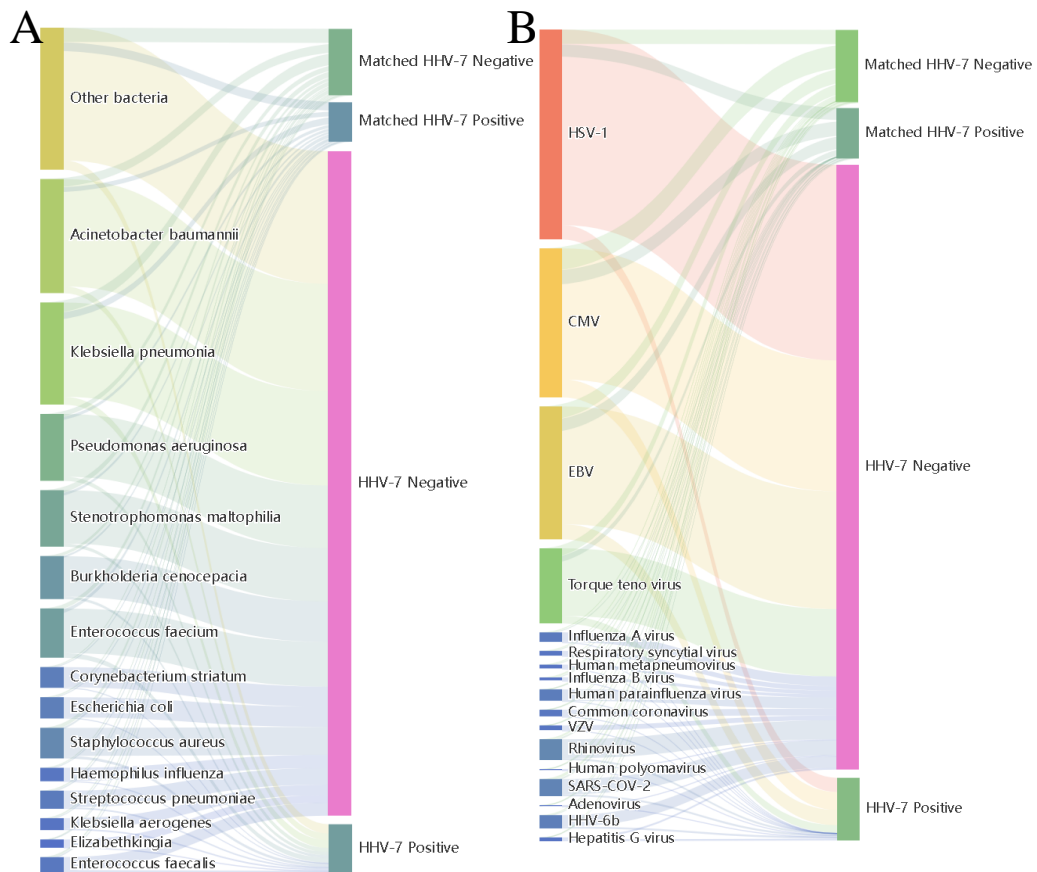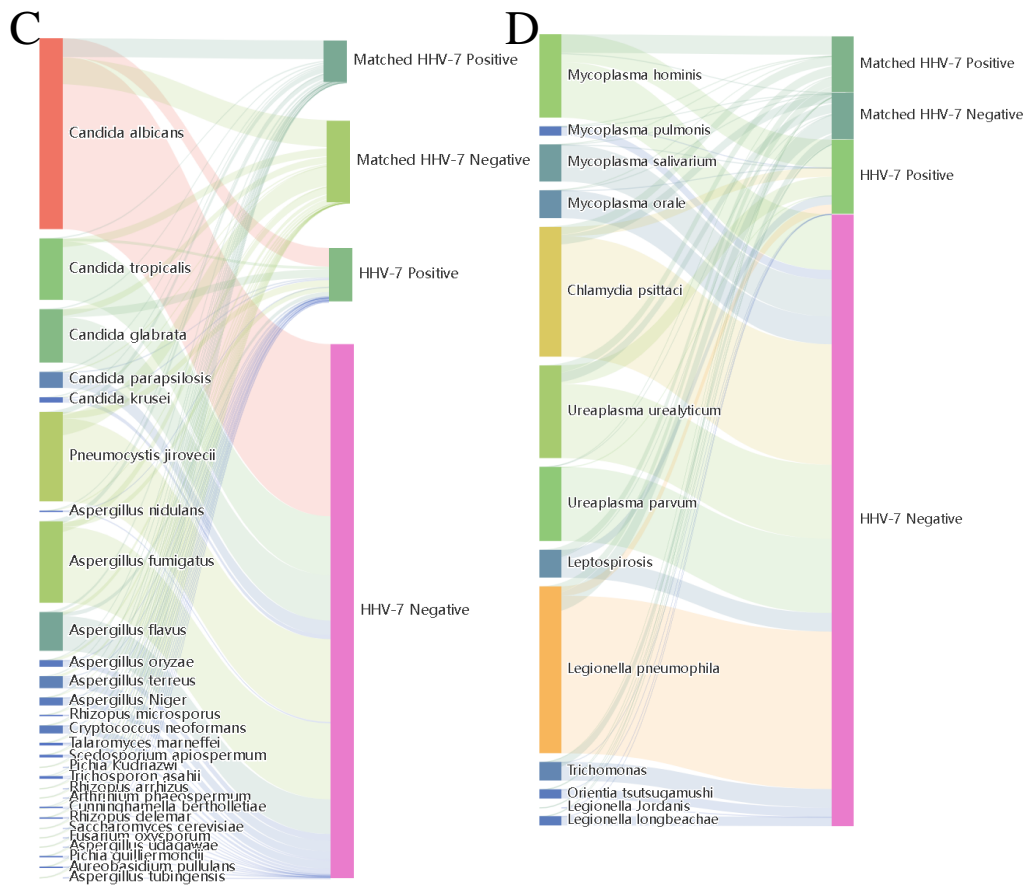

Supplement: Supplementary file 4 — Additional file 4: Fig. S4. Main microbial pathogens including bacteria, virus, fungi, and others detected by mNGS within HHV-7-positive and HHV-7-negative BALF. All data represent the number of times pathogens were detected within different groups, not the number of patients. [file 13054_2023_4530_MOESM4_ESM.pdf]
