# Supplementary material for: Incidence and clinical features of HHV-7 detection in lower respiratory tract in patients with severe pneumonia: a multicenter, retrospective study
Source: Crit Care. 2023 Jun 23;27:248. doi: 10.1186/s13054-023-04530-6 (PMC10290302; doi:10.1186/s13054-023-04530-6)

**A** Kaplan-Meier Estimates of the Probability of Survival

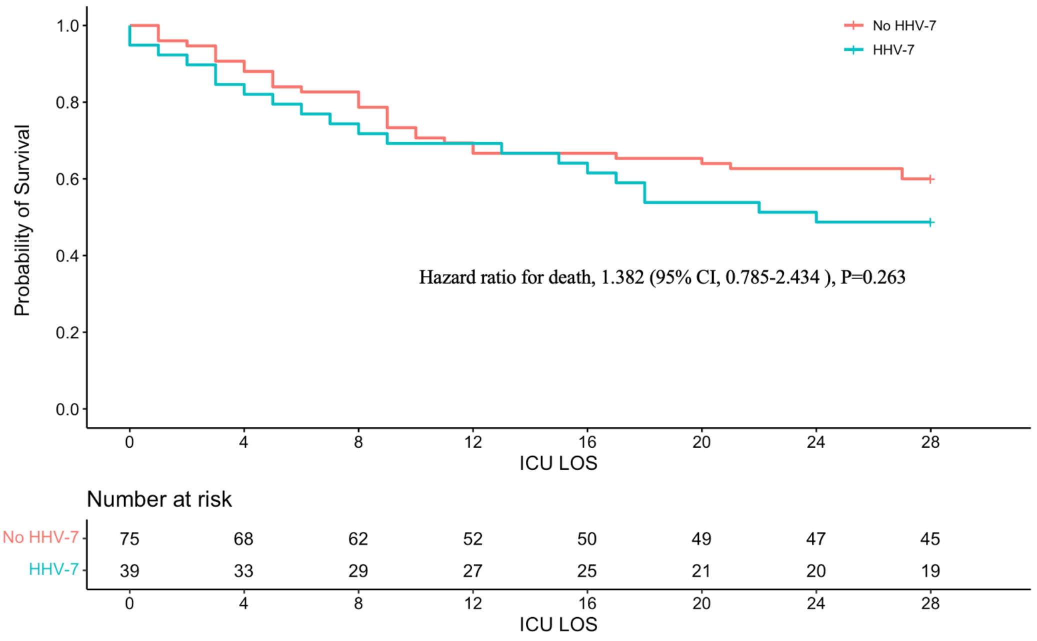


**B** Subgroup Analysis of 28-day Death from Any Cause

| Subgroup | No HHV-7 | HHV-7 | HR (95% CI) | P Value for Interaction |
|---|---|---|---|---|
| | *no. of events / total no. (%)* | | | |
| **Overall** | **30/75(40.0)** | **20/39(51.3)** | **1.382(0.785-2.434)** | **0.263** |
| **Age** | | | | |
| ≤ 65 | 9/33(27.3) | 8/20(40.0) | 1.686(0.650-4.372) | 0.283 |
| > 65 | 21/42(50.0) | 12/19(63.2) | 1.263(0.621-2.569) | 0.519 |
| **Sex** | | | | |
| Female | 6/13(46.2) | 5/8(62.5) | 1.952(0.590-6.389) | 0.275 |
| male | 24/62(38.7) | 15/31(48.4) | 1.260(0.661-2.403) | 0.483 |
| **Immunosuppression** | | | | |
| Yes | 7/20(35.0) | 7/11(63.6) | 2.222(0.776-6.368) | 0.137 |
| No | 23/55(41.8) | 13/28(46.4) | 1.143(0.579-2.258) | 0.699 |
| **Surgical Treatment** | | | | |
| Yes | 5/21(23.8) | 4/11(36.4) | 1.637(0.439-6.101) | 0.463 |
| No | 25/54(46.3) | 16/28(57.1) | 1.336(0.713-2.503) | 0.366 |
| **Other Herpesvirus** | | | | |
| Yes | 14/35(40.0) | 15/27(55.6) | 1.493(0.720-3.097) | 0.281 |
| No | 16/40(40.0) | 5/12(41.7) | 1.158(0.424-3.163) | 0.775 |

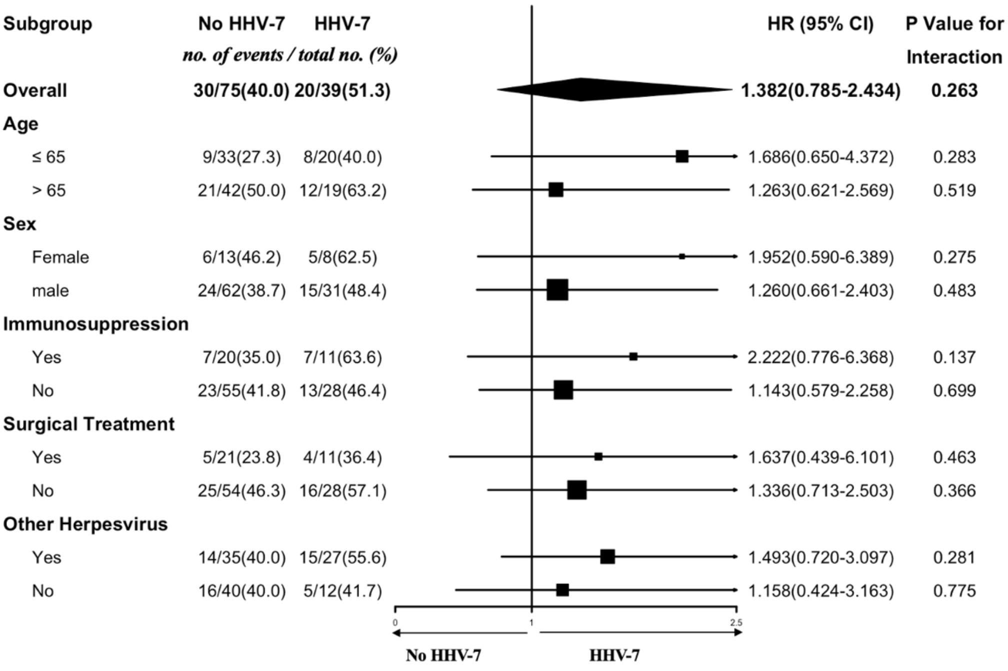

Supplement: Supplementary file 3 — Additional file 3: Fig. S3. Sensitive analysis. [file 13054_2023_4530_MOESM3_ESM.pdf]
